# On the Improvement of Eye Tracking-Based Cognitive Workload Estimation Using Aggregation Functions

**DOI:** 10.3390/s21134542

**Published:** 2021-07-02

**Authors:** Monika Kaczorowska, Paweł Karczmarek, Małgorzata Plechawska-Wójcik, Mikhail Tokovarov

**Affiliations:** Department of Computer Science, Lublin University of Technology, 20-618 Lublin, Poland; m.kaczorowska@pollub.pl (M.K.); p.karczmarek@pollub.pl (P.K.); m.tokovarov@pollub.pl (M.T.)

**Keywords:** aggregation, generalized Choquet integral, fuzzy measure, classical machine learning, cognitive workload

## Abstract

Cognitive workload, being a quantitative measure of mental effort, draws significant interest of researchers, as it allows to monitor the state of mental fatigue. Estimation of cognitive workload becomes especially important for job positions requiring outstanding engagement and responsibility, e.g., air-traffic dispatchers, pilots, car or train drivers. Cognitive workload estimation finds its applications also in the field of education material preparation. It allows to monitor the difficulty degree for specific tasks enabling to adjust the level of education materials to typical abilities of students. In this study, we present the results of research conducted with the goal of examining the influence of various fuzzy or non-fuzzy aggregation functions upon the quality of cognitive workload estimation. Various classic machine learning models were successfully applied to the problem. The results of extensive in-depth experiments with over 2000 aggregation operators shows the applicability of the approach based on the aggregation functions. Moreover, the approach based on aggregation process allows for further improvement of classification results. A wide range of aggregation functions is considered and the results suggest that the combination of classical machine learning models and aggregation methods allows to achieve high quality of cognitive workload level recognition preserving low computational cost.

## 1. Introduction

Cognitive workload is understood as a mental effort necessary to perform a task [1]. It is a non-trivial process useful in explaining mental fatigue and its influence on the brain’s cognitive system performance. Automatic categorizing and classification of cognitive workload levels is a subject of numerous research studies published recently. The classification of cognitive workload can be conducted in two ways: subject-dependent approach [2,3,4] and subject-independent approach [5,6]. Subject-independent approach, being more general, attracts greater attention of the researchers nowadays [7]. The literature review [8] also shows the examples of combined subject-dependent and subject-independent approaches. The most frequent case that can be found in the literature is binary classification problem: distinguishing between low and high levels of cognitive workload [9,10]. Besides the binary approach, papers dealing with three-way classification can be found. In that case, low, medium, and high levels of cognitive workload are considered [6,7,11]. Experiments involving multiclass classification are less common in the cognitive workload research [12,13]. The literature shows the reports of the results obtained with various classifiers, but the most popular among them are Support Vector Machine (SVM) [6,14,15], Linear Discriminant Analysis (LDA) [16], k-Nearest Neighbors (kNN) [11], and Random Forest [6]. In addition to classical recognition models, deep neural network-based approaches such as convolutional deep neural networks [9,17,18] are applied in the cognitive classification process. The reported results of accuracy are in the range of 50–80%. Classification of cognitive workload can be conducted on the basis of electroencephalographic (EEG) data [11], galvanic skin response (GSR) [19], or eye-tracking [20]. In [21,22], the authors use the fuzzy methods to effectively monitor the state of cognitive workload of an Unmanned Aerial Vehicle (UAV) operator. In [23], the authors successfully apply fuzzy cognitive mapping to analyze the pilots’ decision during the flight.

It is worth recalling a few recent results. Fatimah and colleagues [24] published an article on the automatic detection of mental difficulty in arithmetic tasks on the basis of an EEG signal. The authors used a publicly available dataset from MIT PhysioNet, which contains recordings from 36 people. The arithmetic tasks performed by the respondents consisted of subtracting numbers. Based on the number of correct calculations per minute, the performed tasks were divided into two groups: easy and difficult. If the number of incorrect answers was not more than 20%, the tasks were considered easy, otherwise they were considered difficult. For 12 people, the tasks turned out to be easy, and for 24, the tasks were difficult. A two-class classification independent of the examined person was carried out: the main goal was to distinguish between low and high levels of cognitive load. The following classifiers were used: SVM, Decision Tree, and Quadratic Discriminant. Accuracy of the classification was calculated for each electrode separately and for each electrode divided into bands. The best results were achieved for the Quadratic Discriminant classifier, both with and without division into bands for a given electrode [24]. The best accuracy achieved with selected electrode and specific frequency band was as high as 97.2%. In [25], the authors conducted research aimed at detecting various mental states of the pilot such as distraction, workload, fatigue, and normal state. Various biosignals were used in the study: EEG, EKG, EDA, and EEA. Based on the signals collected from eight pilots, a four-class classification was carried out relating to distraction, workload, fatigue, and normal state. The authors presented the results of classification independent of the tested person for various classifiers, among others, for KNN, SVM, sLDA, LSTM, and their own proposed network for the EEG data separately, for the rest of the signals and for the combination of the EEG with the rest of the signals. The best results for the majority of classifiers were obtained for the data considering all signals. For the method proposed by the authors, based on the LSTM, the mean classification score was 85.2% (accuracy). In [26], the authors presented a model based on GALoRIS, thanks to which it is possible to identify high and low cognitive loads. The algorithm selects the features that correspond to low and high loads. The model was tested by the authors on the basis of the cognitive load data associated with driving. EEG data for the experiment were collected while driving the vehicle in the simulator. In addition, the authors used the NASA scale TLX and Instantaneous Self-Assessment (ISA), which enabled the subjective assessment of the individual and the vehicle performance measures (error level). The authors conducted a classification independent of the examined person and tested several classifiers in their research, the best result was achieved for the SVM classifier and was over 96%. Agnola and colleagues [27] dealt with a very interesting topic—the cognitive load in the context of using drones in search-and-rescue (SAR) missions. The authors used a simulator with which three levels of SAR-related cognitive bias were evoked. They used biological signals such as: ECG, skin temperature, respiration. The authors proposed a method of eliminating the extracted features using the following algorithms: eXtreme Gradient Boosting (XGBoost) and Shapley Additive exPlanations (SHAP). Experiment was carried out on 24 people who were asked to perform four activities: baseline, mapping activity, flying activity, flying and mapping activity simultaneously. As in the case of article [26], the authors used the NASA-TLX scale. The article presents the results of classification independent of the tested person, both two-class and three-class using such classifiers as kNN, Logistic regression, LDA, XGBoost, Random Forest. Two-class classification was used for distinguishing between low and high cognitive load. The authors obtained 80.2% accuracy for the two-class classification and 62.9% for the three-class classification using the XGBoost classifier with 24 features. In the paper [28], the authors presented a model that classifies the cognitive load based on the Long Short-Term Memory (LSTM) network and the Filter Bank Common Spatial Pattern (FBCSP) based on EEG data. The authors conducted the two-class classification: arithmetical tasks and rest state; they achieved an accuracy of 87% with this model. In their research, the authors used a publicly available dataset, which contains data from 30 people performing arithmetic tasks.

The poor or unsatisfactory quality of some classifiers in various fields of application can be compensated by the use of appropriate operators aggregating the classification results returned by these classifiers separately or on the basis of an information fusion at the stage of the data preprocessing. The former way of finding the final ranking of classification results is intuitively appealing and typical for many fields of application such as sport competitions, risk analysis, decision-making, etc. These aggregation functions or operators are described in detail in many monographs [29,30,31,32,33,34] and papers [35,36,37]. In particular, typical classes of aggregation operators are means, triangular norms [38,39], ordinary weighted averaging operators [35,40], Choquet integral, and its generalizations [41,42,43,44,45,46,47] called pre-aggregation functions, etc. Comprehensive experimental studies, in particular, on an applications of aggregation operators and generalizations of Choquet integral to the face recognition problems were presented in [44,46,48], respectively.

The main goal of this study is to improve the results of eye activity and user performance-based cognitive workload level classification with the use of aggregation methods. For this purpose, we test and compare over 1000 classic aggregation operators and over 1000 pre-aggregation operators (so called generalized Choquet integrals) to determine the best one. The set of aggregation operators utilized in a series of thorough numerical experiments is built on the basis of above-mentioned monographs [29,30,31,32,33,34] and selected papers. We list the best aggregation functions and discuss the accuracies obtained for the typical classifiers such as Decision Tree, k-Nearest Neighbors, etc. The dataset used in the classification study contains eye-tracking and user performance data taken from 29 participants in the study of solving the computerized version of Digit Symbol Substitution Test (DSST).

The rest of the paper is structured as follows. Section 2 presents the description of the experiment procedure with detailed explanation of eyetracking-related aspects and data processing methods applied. Section 3 presents the utilized aggregation functions. Section 4 contains the presentation of the results obtained with individual classifiers as well as the recognition rates achieved with application of the presented aggregation functions. Section 5 concludes the paper and presents the future work directions.

## 2. Eyetracking

### 2.1. Research Procedure

**Figure 1:**
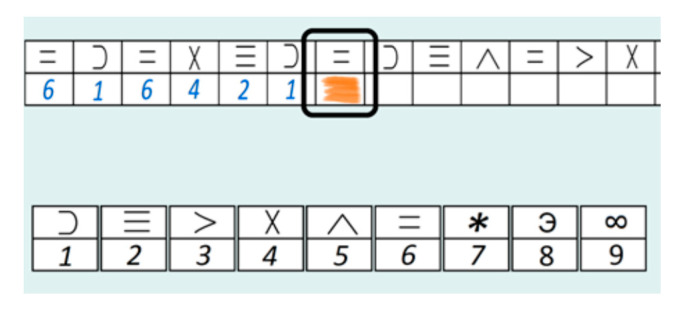
The interface of the application.

The dataset containing eye activity and user performance data was gathered using the computerized version of the DSST test [49] developed for the purpose of this study. The idea of DSST test is to match displayed symbols to particular digits according to a key presented continuously on the screen (Figure 1). In the study, participants were asked to assign subsequent symbols to digits within the specified time. Symbols were generated randomly and with repetition. Participants were instructed to perform as many correct matches as possible within defined time. The time of single trial and the number of different symbols to be displayed were defined in the application settings. For the purpose of the study, three DSST parts were prepared; each of them corresponded to one cognitive workload level in the further analysis. Part 1 corresponding to the low level of cognitive workload, contained four different symbols, and the time was set to 90 s. Part 2 related to the medium level of cognitive workload, covered nine different symbols, and the time was also set to 90 s. Part 3 defined for the hard level of cognitive workload, covered nine different symbols, and the time was extended to 180 s. In all parts, participants were asked to perform as many matchings of subsequent symbols to digits as possible (in defined time). They were also instructed to perform matches as fast as possible. The settings were defined empirically based on the preliminary pilotage study. Each participant of the case study was asked to perform all three DSST parts. The experiment was preceded by short trial to familiarize participants with the application.

The experiment was performed in a laboratory room illuminated with standard fluorescent light. The eye activity data were gathered using Tobii Pro TX300 screen-based eye tracker (Tobii AB, Stockholm, Sweden), which was built into a monitor (23′′ TFT monitor, 60 Hz) connected to the computer. Data were registered with the frequency of 300 Hz. Tobii Studio 3.2 software was used to design the experiment and export data. Each session was preceded by the 9-point calibration procedure.

Eye activities gathered in the experiment were related to such measures as fixations, saccades, blinks, and pupil size. Fixations are understood as the period of uptaking visual information, during which a participant holds eyes stable in a particular position. Saccades are understood as the rapid eye movement occurring between fixations. The dataset covered 20 selected features related to fixations (total number of fixations, mean duration of fixation, standard deviation of fixation duration, maximum fixation duration, minimum fixation duration), saccades (total number of saccades, mean duration of saccades, mean amplitude of saccades, standard deviation of saccade amplitude, maximum saccade amplitude, minimum saccade amplitude), blinks (total number of blinks, mean of blink duration), and pupillary response (mean of left pupil diameter, mean of right pupil diameter, standard deviation of left pupil diameter, standard deviation of right pupil diameter). Moreover, data related to DSST test results, i.e., number of errors, mean response time, and response number, were also included.

The experiment was conducted on a homogeneous group of 30 participants: 24 males, six females aged 20 to 24 (mean = 20.61 years, std. dev. = 1.54) recruited among healthy students of the BSc degree in computer science. The participants reported to have normal/corrected to normal vision and they were not under strong medicines. As the acceptable level of registered data activity was set to 90%, data from one participant were discarded from the further analysis due to their poor quality.

### 2.2. Data Processing

The data processing procedure was composed of six steps: data acquisition, data synchronization, feature extraction, feature normalization, feature selection, training, and testing classification models. The raw data were generated in the form of six files per single participant (two files (eyetracking data and DSST results) for each of three DSST parts). Owing to that fact, a synchronization procedure was needed. Finally, 87 observations were included in the output dataset (three observations representing three cognitive workload levels per single participant). In the feature extraction procedure, twenty independent features were obtained. Feature normalization was also performed to guarantee a uniform feature scale.

The ANOVA analysis was performed for 17 features. The K-S test and Levene test were previously performed to check assumptions of normality of distribution and equality of variance. In this process, three of 20 features (mean duration of saccades, minimum saccade amplitude, and mean of blink duration) were discarded from further analysis. The ANOVA analysis revealed 10 significant features (*p*-value 0.05), which were applied in classification process. The Tukey’s HSD post-hoc test was applied in order to identify the pairs of DSST parts which differed significantly. Table 1 presents significant results (*p*-value < 0.05) of the ANOVA analysis. 

The classification procedure was focused on assigning observations into one of the three classes: low, medium, and high level of cognitive workload. Various classification methods such as SVM, kNN, Decision Tree, Random Forest, Multilayer Perceptron (MLP), and Logistic Regression were applied. As the classification was performed using a subject-independent approach, the division into train and test datasets was done in such a way that a single participant could be used only in one dataset. The test dataset covered data from six participants, which corresponded to approximately 20% of the input dataset. 

In order to investigate the influence of particular features of classification process, feature importance ranking was generated. Table 2 presents the features ranked with respect to their importance for classifying procedure. The results were obtained based on Logistic Regression model. 

## 3. Aggregation of Classifiers

Let us recall the most important properties of aggregation operators. Aggregation function *p*: ${\left[0,\;1\right]}^{n}\to \left[0,\;1\right]$ is, in general, defined as an operator fulfilling the following conditions:

(1) p(0,0,…,0)=0,p(1,1,…,1)=1

and

(2) $$\forall x,y\in {\left[0,\;1\right]}^{n}x\leq y\Rightarrow P\left(x\right)\leq p\left(y\right)$$

It means that it preserves bounds and monotonicity [31]. Examples are various means or Ordinary Weighted Averaging (OWA) operators [40]. One of the most important and intensively developed aggregation operators is the Choquet integral. To define this integral, we have to recall the properties of fuzzy measure. If *X* is a set then $Q\left(X\right)=2^{X}$ is its subsets family. Then a function *g* fulfilling the conditions

(3) g(∅)=0

(4) g(X)=1

(5) g(A)≤g(β),  A⊂B,  A,B∈Q(X)

(6) $$\underset{n\to \infty }{lim}g\left(A_{n}\right)=g\left(\underset{n\to \infty }{lim}A_{n}\right)$$

where {*A_n_*}; *n* = 1, 2, …, denotes an increasing sequence is called fuzzy measure. Note that the Sugeno λ-fuzzy measure is a typical example of fuzzy measure class of functions. Recall that it satisfies

(7) g(A∪B)=g(A)+g(B)+λg(A)g(B)

for *λ* > −1. Here, *A* and *B* are not overlapped. Moreover,

(8) $$g\left(A_{i+1}\right)=g\left(A_{i}\right)+g_{i+1}+\lambda g\left(A_{i}\right)$$

where *A_i_* = {*x*_1_, …, *x_n_*}, *A_i_*_+1_ = {*x*_1_, …, *x_n+_*_1_}. To simplify one writes

(9) $$g_{i}=g\left(\left\{x_{i}\right\}\right)\;i=1,\cdots ,n$$

Let *h(x)* be a function and let *h(x_i_)*, *i* = 1, …, *n*; be ordered in a non-increasing manner. Moreover, let *h(x_n_*_+1_*)* = 0. Then the Choquet integral is

(10) $$CH=\overset{n}{\underset{i=1}{\sum }}\left(h\left(x_{i}\right)-h\left(x_{i+1}\right)g\left(A_{i}\right)\right)$$

An interesting generalization for this function is [46,48]

(11) $$C_{MMin}\left(x\right)=\overset{n}{\underset{i=1}{\sum }}M\left(\min\left(h\left(x_{i}\right),g\left(A_{i}\right)\right)-\min\left(h\left(x_{i+1}\right),g\left(A_{i}\right)\right)\right)$$

or

(12) $$C_{MinM}\left(x\right)=\overset{n}{\underset{i=1}{\sum }}\left(\min\left(M\left(h\left(x_{i}\right),g\left(A_{i}\right)\right),g\left(A_{i}\right)\right)-\min\left(M\left(h\left(x_{i+1}\right),g\left(A_{i}\right)\right),g\left(A_{i}\right)\right)\right)$$

Here, *M* can be any t-norm, see [43,44]. 

**Figure 2:**
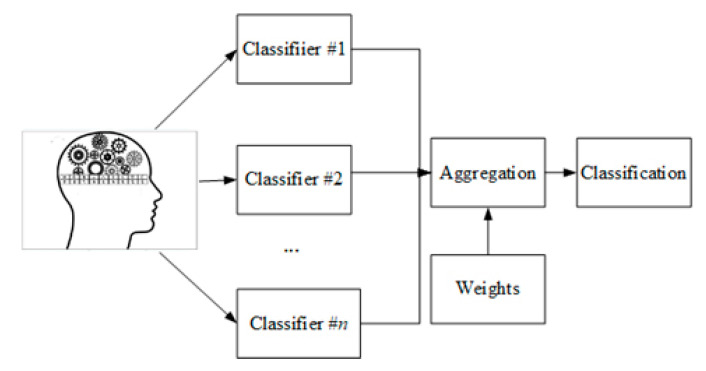
A general aggregation scheme.

A general model of aggregation processing is presented in Figure 2. The data are classified separately by various classifiers. Next, on a basis of weights, which can be obtained from experts or on a basis of accuracy of individual classifiers, the results are aggregated using a proper aggregation operator.

## 4. Experimental Results

### 4.1. Individual Clssifiers

Several classic machine learning models were tested in the first stage of numerical experiments. The following classifiers were applied: SVMs with various kernels, namely linear, quadratic, and cubic one, Logistic Regression, k-Nearest Neighbors, Decision Tree, Random Forest, Multilayer Perceptron (MLP). Due to the fact that the test sample was balanced, accuracy can be an appropriate classification quality metric. Table 3 shows the mean values of accuracy obtained for various classifiers achieved for both datasets: the dataset containing all 20 features and the dataset containing 10 selected features. It can be noticed from the results, the best classification model allowed to achieve the accuracy reaching the level of 96%. The results show that the classifier accuracy for dataset with selected features are slightly better than the results obtained for all features.

**Figure 3:**
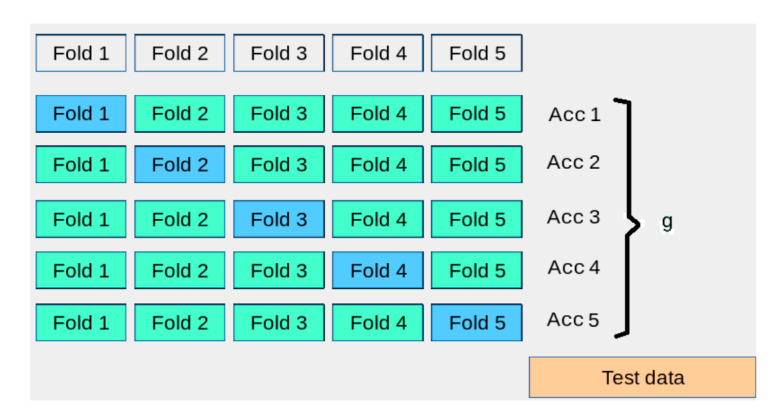
The idea of fuzzy measure generation through the process of cross validation.

Another important aspect worth noting here is the procedure of fuzzy measure density values generation. Several methods of fuzzy measure generation can be used: expert assumption, optimization, and, finally the heuristic one. In our research, we use the heuristic based on cross validation. In order to produce a density measure for a classifier, we run *n*-fold cross validation on the training set. As the result we obtain *n* values of accuracy. The mean of cross validation accuracy is considered as the fuzzy measure *g_i_* of the *i*-th classifier. The fuzzy measures can be interpreted as the degree of trust (or simply weights or level of importance) to a separate classifier’s predictions. Figure 3 illustrates the approach.

### 4.2. Aggregation of Classifiers

**Figure 4:**
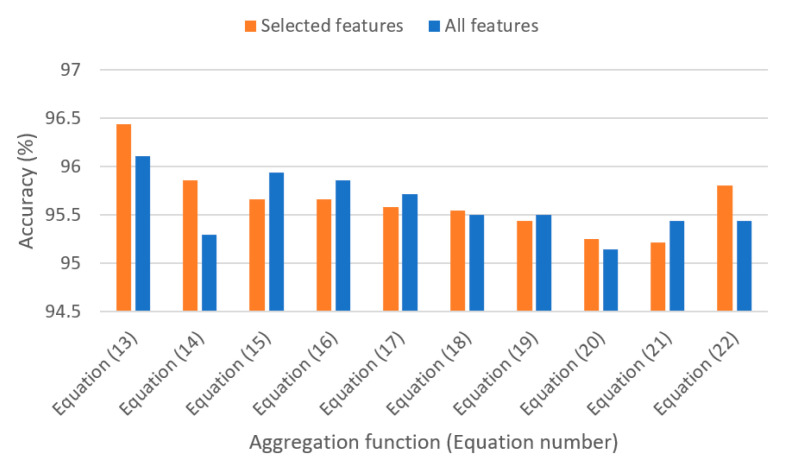
Comparison of accuracies achieved with top aggregation functions on complete feature set and for selected features.

Here, we present the best functions serving as aggregation operators for the classifiers listed in the previous subsection, i.e., Cubic SVM, Decision Tree, k-Nearest Neighbor, Linear SVM, Logistic Regression, Multilayer Perceptron, Quadratic SVM, and Random Forest. In the cases where it is needed to feed the aggregation algorithm with weights, they were found on a basis of specific classifiers’ accuracy by performing cross validation on training data. For instance, to determine fuzzy measure densities *g_i_*, see Equation (9). The values being the inputs to the aggregation functions are the probabilities of belonging to the three considered classes. Depending on the number of arguments of the specific aggregation function, these values are either provided to a single function or transitive. The latter case is considered when the function has only two arguments. In the validation stage, we considered 200 repetitions, each including tests on 18 validation observations for which we have obtained the probabilities of belonging to the three classes. Let us now discuss the best aggregation operators from over 2000 aggregation operators and so-called pre-aggregation functions (generalized Choquet integrals), see papers [43,45]. The source of the functions were various examples or our own modifications of the functions comprehensively described in [29,31,34,38,50,51] and other books and papers. In the rest of the section, we present the results obtained with particular aggregation operators: both for complete feature set and for selected 10 features. The results are provided in the following format: “selected features result” (“complete feature set result”). The summary of the results is presented on Figure 4.

The best result was obtained with a so-called generalized form of Choquet integral [34], i.e.,

(13) $$L\left(x,y\right)=\{\begin{array}{c} ax+\left(1-Q\right)y\;\;for\;x\geq y\\ \left(1-b\right)x+by\;\;otherwise \end{array}\;$$

where *x* ≥ 0, *y* ≥ 0, *a*, *b* ϵ [0, 1]. It gave the accuracy 96.44% (96.11%) for various parameters of a and b, for instance *a* = 0.01, *b* = 0.99. Other selected values of these parameters resulted in correct recognition rates on a slightly lower level. Here, it is worth stressing that the name of the function (12) can be misleading since it is not typical Choquet integral discussed in the previous section, see Equation (10). 

The next function producing satisfying results 95.86% (95.3%) is a so-called weighted aggregation function of the form [34]

(14) $$A\left(x_{1},\ldots ,x_{n}\right)=\frac{\prod_{i=1}^{n}\left(1+w_{i}x_{i}\right)-\prod_{i=1}^{n}\left(1-w_{i}x_{i}\right)}{\prod_{i=1}^{n}\left(1+w_{i}x_{i}\right)+\prod_{i=1}^{n}\left(1-w_{i}x_{i}\right)}$$

where the values of $w_{i}$’s are the individual classifiers’ accuracies.

The next function, which produces highly satisfying results, is Stolarsky mean [34], [52]

(15) $$M_{s}\left(x,y\right)=\{\begin{array}{c} {\left(\frac{x^{r}-y^{r}}{r\left(x-y\right)}\right)}^{\frac{1}{r-1}}if\;x\neq y\\ x^{}\;if\;x=y \end{array}\;$$

where *r* ≠ 0. In this case, the resulting recognition rate is 95.66% (95.94%). For *r* = 2. The next interesting function is an associative function proposed in [29], namely

(16) $$C\left(x,y\right)=\frac{1}{2}W\left(x,y\right)+M\left(x,y\right)\;$$

where

W(x,y)=max(x+y−1,0) 

and

$$M\left(x,y\right)=\frac{x+y}{2}$$

with 95.66% (95.86%) accuracy. A so-called SP-based bivariate symmetric sum [31]

(17) $$f\left(x,y\right)=\frac{x+y-xy}{1+x+y-2xy}$$

produced the recognition rate of the level of 95.58% (95.72%). The function of the form

(18) $$f\left(x,y\right)=2^{\log\left(1+x\right)\log\left(1+y\right)/{\left(\log2\right)}^{2}}$$

gave 95.55% (95.5%) recognition rate. The accuracy 95.44% (95.5%) was obtained with an application of a function of the form

(19) $$f\left(x,y\right)=\frac{x+y}{2}$$

but if x∈ [0.5, 0.7) the value of x is substituted by 0.5. The same is done with y∈ [0.5, 0.7). Good results are also obtained with a so-called 1-Lipschitzian aggregation function (Bertino copula) [34] (p. 271)

(20) $$f\left(x,y\right)=\{\begin{array}{c} {\left(\mathrm{Min}\left(x,y\right)\right)}^{2},\;\mathrm{if}\;x\leq y\\ {\left(\mathrm{Max}\left(x,y\right)\right)}^{2}-\left|x-y\right|,\;\mathrm{otherwise} \end{array}\;$$

returns 95.25% (95.15%) accuracy. Finally, Sugeno integral [34,50] and max-based bivariate symmetric sum [31], i.e.,

(21) $$f\left(x,y\right)=\frac{\max\left(x,y\right)}{1+\left|x-y\right|}$$

yielded 95.22% (95.44%) recognition rate.

Very good results can also be obtained with the generalization of the Choquet integral of the form (11) and (12). The function *M* standing under the integral sign was

(22) $$M\left(x,y\right)={\left(\ln\left(e^{x^{-\alpha }}+\ln\left(e^{y^{-\alpha }}-e\right)\right)\right)}^{-\frac{1}{\alpha }}\;$$

for α>0. Its value α=3.3 gave the maximal recognition rate at the level of 95.81% (95.44%).

Here, it is worth stressing that also the results at satisfying level were obtained using various fuzzy integrals, most of the pre-aggregation functions or generalized aggregation functions discussed in [38], median or weighted median, scoring or weighted scoring, quadratic mean, and a few versions of ordinary weighted averaging functions (OWA). Interestingly, aggregation operators can improve recognition rate in more noticeable way for the data without extended feature selection.

Figure 4 presents the ranking of the best operators among the tested aggregation functions. The results show that their application affects the quality of classification in a favorable way. The best result, achieved with a generalized form of Choquet integral function, is more than 1.2 percentage point higher for complete feature set and 0.2 percentage point higher for selected features compared to the best individual classifier (Logistic Regression and Random Forest).

## 5. Discussion

The aim of the study was to improve the result of multiple cognitive workload level classification based on eye activity and user performance. The original classification procedure covering three class classification using classical methods such as SVM, kNN, Decision Tree, Random Forest, MLP, and Logistic Regression was the input to the aggregation functions. In the study, many aggregation and pre-aggregation operators published in the core literature monographs were compared in order to find the best model suitable for classification of cognitive workload level. The results show that using various classification models in combination with an aggregation function allows further improvement of recognition rate by applying the knowledge cumulated in the parameters of the trained models. 

The original dataset covering eye-tracking and user performance data was gathered in a study of three parts of the computerized version of DSST test (Digit Symbol Substitution Test). Classification was performed with the interpretable machine learning model in order to regard the most valuable features. Eye-tracking features, in general, have been already proved to be useful in cognitive workload analysis also due to the fact that it is a non-invasive sourced, natural type of response obtained without additional activity or training. What is more, the classification was performed as subject-independent in order to distinguish classes regardless of such conditions as the age of an examined person, his/her habits, or testing period. The best original classification results achieved 96%. It is worth noting that the tests were performed on a homogeneous group of healthy people with similar age and educational level.

The study presented in the paper proved that applying aggregation methods enables to increase the classification result by more than 1 percentage point. Detailed results show that there were several aggregation functions that enabled achieving the highest results (presented in the paper are the top ten functions as Equations (13)–(22)). 

Classification results, both individual and with aggregation, prove that the time and difficulty level of performed tasks have a systematic influence on user performance, pupillary and eye movements. The results show that there is a relation between the participants’ engagement combined with cognitive state and eye activity. The most important features in the study are these related to the user performance and the intensity of eye movement. It indicates that fixation and saccade-related features (mean saccade amplitude, standard deviation of fixation duration, total number of fixations and saccades) as well as response-related features (mean response time, response number) reflect the degree of attention during the tasks performance. However, further results are needed to investigate additional factors such as types of tasks, participant profiles or their initial mental state. What is more, it is worth to consider the mental abilities of each single participant. Such information might help to adjust the cognitive workload to a particular participant. This might be measured with dedicated models or surveys (e.g., NASA-TLX scale, the Rasch and strain–stress model), although such tools are based on subjective assessment. 

A broad set of pre-aggregation and aggregation operators was analyzed in the study in order to find the ones that fit the best to the analyzed problem. The detailed results show that the classification accuracy was improved.

In the case study, two approaches were applied. The first one was based on classification considering original 20 features whereas the second one covered 10 features chosen in statistical analysis. The individual classification results for both approaches differ slightly, although the results for smaller number of features occurred to be better. Results for both approaches were further processed in order to apply pre-aggregation and aggregation operators. The best results for both approaches were achieved for the generalized Choquet integral. This operator enabled to improve the classification results by as much as 1.2 percentage point for all features-based approach compared to the best classification model. The same operator proved to be efficient also in case of a smaller feature number approach, although the improvement was not as high. It was Random Forest that occurred to be the best among the classical classifiers for both approaches. Additionally, Logistic Regression gave similar results for the second approach. These results confirm usefulness of the generalized Choquet integral found in research over classification performance. The results prove that the application of pre-aggregation and aggregation operators is useful especially in case of applying the basic feature selection. Aggregation functions might give better improvement in case of weaker initial individual classification results.

Future work is planned to include the experiments on a broader dataset, collected from a higher number of participants. The authors also consider analysis of a higher number of cognitive workload levels. As further development of the topic, it is planned to include self-report tools of detecting mental illness such as depression or anxiety symptoms in our future work.

## Figures and Tables

**Table 1:** The results of one-way ANOVA analysis.

	ANOVA	Post-Hoc Test		
Features	*p*-Value	*p*-Value Class 1–Class 2	*p*-Value Class 1–Class 3	*p*-Value Class 2–Class 3
response number	<0.001	<0.001	<0.001	<0.001
mean response time	<0.001	<0.001	<0.001	0.69
total number of fixations	<0.001	0.36	<0.001	<0.001
standard deviation of fixation duration	0.002	0.003	0.008	0.95
maximum fixation duration	0.009	0.011	0.04	0.87
total number of saccades	<0.001	0.56	<0.001	<0.001
maximum saccade amplitude	0.002	0.41	0.001	0.046
mean saccade amplitude	<0.001	<0.001	0.09	<0.001
total number of blinks	0.015	0.99	0.003	0.003
standard deviation of pupil diameter (left)	0.005	0.016	0.012	0.99

**Table 2:** Separate class feature rankings together with weights obtained by interpreting the weights of the Logistic Regression model.

No.	Low	Medium	High
1	mean saccade amplitude (1.0)	mean response time (1.0)	response number (1.0)
2	mean response time (0.95)	response number (0.65)	total number of fixations (0.95)
3	standard deviation of fixation duration (0.6)	mean saccade amplitude (0.63)	total number of saccades (0.95)
4	total number of fixations (0.53)	standard deviation of fixation duration (0.62)	mean saccade amplitude (0.22)
5	total number of saccades (0.52)	total number of fixations (0.6)	maximum saccade amplitude (0.19)
6	response number (0.27)	total number of saccades (0.55)	mean response time (0.18)
7	standard deviation of pupil diameter (left) (0.17)	maximum fixation duration (0.28)	maximum fixation duration (0.15)
8	maximum fixation duration (0.16)	maximum saccade amplitude (0.15)	total number of blinks (0.1)
9	maximum saccade amplitude (0.5)	total number of blinks (0.09)	standard deviation of pupil diameter (left) (0.09)
10	total number of blinks (0.1)	standard deviation of pupil diameter (left) (0.05)	standard deviation of fixation duration (0.08)

**Table 3:** Accuracies obtained with separate classifiers.

Model	Accuracy (%) for 10 Selected Features	Accuracy (%) for All Features
SVM(Linear)	94.75	93.11
SVM(Quadratic)	84.47	78.28
SVM(Cubic)	92.36	89.47
Logistic Regression	96.22	94.67
kNN	93.78	89.61
Decision Tree	90.39	90.11
Random Forest	96.22	94.89
MLP	93.53	89.56

## Data Availability

The data presented in this study are available on request from the corresponding author.
